# 

**Published:** 1887-07

**Authors:** 


### THE AMERICAN DENTAL ASSOCIATION.

For the information of members and delegates attending the meeting of the Association at Niagara Falls, Tuesday, August 2nd, I will state that the Casino has been engaged for four days. The following are the hotel rates:

AMERICAN DENTAL ASSOCIATION—HOTEL RATES AT NIAGARA.—*International Hotel*—Accommodates 300 guests. Special rates to members and families, $3. *Prospect Park House*—Accommodates 100 guests. Special rate if double beds are occupied, $2.50. *Hotel Atlantique* (German)—Accommodates 50 guests. Special rate if double beds are occupied, $2. *Cataract House*—Accommodates 300 guests. Regular rate (no reduction), $4. *Hotel Kaltenbach*, (German)—Accommodates 50 guests. Regular rate (no reduction), $3. *Niagara House*—Accommodates 75 guests. Regular rate, (no reduction), $2. *Temperance House*—Accommodates 75 guests. Regular rate (no reduction), $2.

The railroads have given reduced rates as follows: All roads comprising the Trunk Line Association, the Southern Passenger Association, the Central Traffic Association and the Western States Passenger Association will carry delegates, members and their families for one and one-third fare for the round trip. Certificates must be signed before returning (by Dr. Geo. H. Cushing, the Secretary), in order to get the reduced rate. Pay your full fare going to Niagara, and take a receipt from the ticket agent, at the starting point. Tickets are good, going three days before the meeting and returning three days after the meeting.

Blank certificates may be obtained from Dr. S. A. Freeman, 14 Court Street, Buffalo, N. Y., and Dr. L. D. Shepard, 100 Boylston Street, Boston, for all points east of Buffalo and Niagara Falls.

Dr. W. C. Wardlaw, Augusta, Georgia, will furnish blank certificates for all points south of the Ohio river, and the south-west.

Dr. George H. Cushing, 96 State Street, Chicago, and Dr. A. W. Harlan, 70 Dearborn Street, Chicago, will furnish certificates for all western points and Indiana, Ohio, Michigan and all points covered by the Central Traffic Association's lines.

The Committee has had great difficulty in getting reduced rates, and it is hoped that as many members, delegates and their families will attend the meeting as can do so, to make of this a notable event in the history of dentistry. Turn out in full force, and bring new inventions, appliances and anything of interest to those assembled.

Yours truly,

A. W. HARLAN,

*Chairman Executive Committee.*

### *To Readers and Correspondents!*

Communications intended for publication, and exchange journals and papers should be addressed to the EDITOR OF THE AMERICAN JOURNAL OF DENTAL SCIENCE, 845 N. Eutaw St., Baltimore, Md. All letters relating to the business of the JOURNAL, or containing cash remittances, to be directed to Messrs. SNOWDEN & COWMAN. Articles for publication should be received by the editor at least three weeks previous to the first month on which the number in which it is designed for them to appear, is issued.

☞ The AMERICAN JOURNAL OF DENTAL SCIENCE will contain fifty pages of reading matter in each number, making a volume of about *Six Hundred* pages,

EXCLUSIVE OF ADVERTISEMENTS.

